# Population genetics of the understory fishtail palm *Chamaedorea ernesti-augusti *in Belize: high genetic connectivity with local differentiation

**DOI:** 10.1186/1471-2156-10-65

**Published:** 2009-10-09

**Authors:** Angélica Cibrián-Jaramillo, Christine D Bacon, Nancy C Garwood, Richard M Bateman, Meredith M Thomas, Steve Russell, C Donovan Bailey, William J Hahn, Samuel GM Bridgewater, Rob DeSalle

**Affiliations:** 1 Department of Ecology, Evolution and Environmental Biology, Columbia University, 1200 Amsterdam Avenue, Mail Code 5557, New York, NY 10027, USA; 2 Department of Biology, New Mexico State University, P.O. Box 30001 MSC 3AF, Las Cruces, NM 88003, USA; 3 Department of Plant Biology Life Science II, Southern Illinois University, Mail Code 6509, 1125 Lincoln Drive, Carbondale, IL 62901, USA; 4 Jodrell Laboratory, Royal Botanic Gardens Kew, Richmond, Surrey, TW9 3DS, UK; 5 Department of Plant Sciences, University of Cambridge, Downing Street, Cambridge, CB2 3EA, UK; 6 Natural History Museum, Cromwell Road, London, SW7 5BD, UK; 7 Department of Biology, Georgetown University, 37th & O Streets NW, Washington, DC 20057, USA; 8 Sackler Institute for Comparative Genomics, American Museum of Natural History, Central Park West at 79th Street, New York, NY 10024, USA; 9 Department of Biology, Colorado State University, Campus Delivery 1878, Fort Collins, CO 80523, USA; 10 Current address: Plant Genomics Laboratory, The New York Botanical Garden, 200th Street and Kazimiroff Boulevard, Bronx, NY 10458-5126, USA

## Abstract

**Background:**

Developing a greater understanding of population genetic structure in lowland tropical plant species is highly relevant to our knowledge of increasingly fragmented forests and to the conservation of threatened species. Specific studies are particularly needed for taxa whose population dynamics are further impacted by human harvesting practices. One such case is the fishtail or xaté palm (*Chamaedorea ernesti-augusti*) of Central America, whose wild-collected leaves are becoming progressively more important to the global ornamental industry. We use microsatellite markers to describe the population genetics of this species in Belize and test the effects of climate change and deforestation on its recent and historical effective population size.

**Results:**

We found high levels of inbreeding coupled with moderate or high allelic diversity within populations. Overall high gene flow was observed, with a north and south gradient and ongoing differentiation at smaller spatial scales. Immigration rates among populations were more difficult to discern, with minimal evidence for isolation by distance. We infer a tenfold reduction in effective population size *ca*. 10,000 years ago, but fail to detect changes attributable to Mayan or contemporary deforestation.

**Conclusion:**

Populations of *C. ernesti-augusti *are genetically heterogeneous demes at a local spatial scale, but are widely connected at a regional level in Belize. We suggest that the inferred patterns in population genetic structure are the result of the colonization of this species into Belize following expansion of humid forests in combination with demographic and mating patterns. Within populations, we hypothesize that low aggregated population density over large areas, short distance pollen dispersal via thrips, low adult survival, and low fruiting combined with early flowering may contribute towards local inbreeding via genetic drift. Relatively high levels of regional connectivity are likely the result of animal-mediated long-distance seed dispersal. The greatest present threat to the species is the potential onset of inbreeding depression as the result of increased human harvesting activities. Future genetic studies in understory palms should focus on both fine-scale and landscape-level genetic structure.

## Background

In plants, tools from molecular ecology and population genetics have been used to characterize genetic patterns that result from geographic and biological barriers to pollen and seed dispersal, and to investigate their significance in the evolutionary history of a species [1-5]. Despite critical insights into many plant species, most studies have focused on temperate trees, followed by tropical canopy trees and temperate herbs. In contrast, palms (Arecaceae) with 2,522 species and iconic significance throughout the tropics [6], have been largely overlooked in this context.

Within palms, understory species are the least represented. Only a handful of studies have focused on the basic population genetics of this group [7-11]. Understory palms are one of the most ecologically and economically important plant groups throughout tropical forests, where human-induced landscape changes occur at an increasing rate [12,13]. Hamrick [14] suggested that most trees have enough intrinsic genetic variation and mechanisms to maintain propagule movement in order to be resilient to habitat changes. While this might be the case for most forest canopy species [15], evidence is less clear for understory taxa, which differ from canopy species in biological and demographic factors. For instance, understory palms have small life forms, smaller seeds than canopy palms, striking phenotypic plasticity, generally narrow distributions along microenvironments, and most notably, variation in population densities across geographic areas [16-21]. They are sensitive to processes that occur at local spatial scales, such as changes in elevation or light within a single mountain slope [22-25]. All of these factors are important determinants of the genetic variation and structure within and among plant populations [1,2,26]. It remains unclear whether theoretical expectations of genetic erosion indeed occur after forest fragmentation in understory palms, or whether other factors, such as changes in demography due to overharvesting or localized ecological degradation, may be more important for the long-term survival of these important species [12].

**Figure 1:**
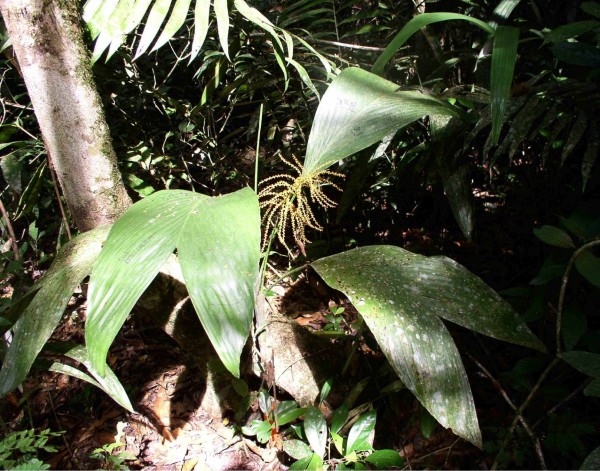
***Chamaedorea ernesti-augusti *male adult plant in the understory forest of Chiquibul, Belize**. Photograph reproduced with permission from H. Porter-Morgan.

The genus *Chamaedorea *is one of the largest and most species-rich of the Neotropical Arecaceae, with *ca*. 96 species widely distributed in the Americas [27,28]. *Chamaedorea ernesti-augusti *is a >5 m tall dioecious perennial species that can live for up to 15 years [29] (Figure 1). They flower once per year and have a thrip-mediated pollination system (via *Brooksithrips chamaedoreae*: Thysanoptera) [30]. Its subglobose, aromatic, black fruits and red rachises imply a combination of gravity and animal dispersal, possibly squirrels and ground-foraging birds [31]. The distribution of this species encompasses tropical evergreen forests in southern Mexico, Guatemala, Belize, and Honduras [32-34].

In recent years, *C. ernesti-augusti *has gained economic importance as an important Non-Timber Forest Product (NTFP) in Central America [35-37]. The leaves of this species, known as xaté or fishtail palm, are harvested and exported into the international floral industry, generating increasingly important revenue [37]. The combination of over-harvesting and habitat loss has led to populations in this region becoming progressively more vulnerable [38,39].

**Figure 2:**
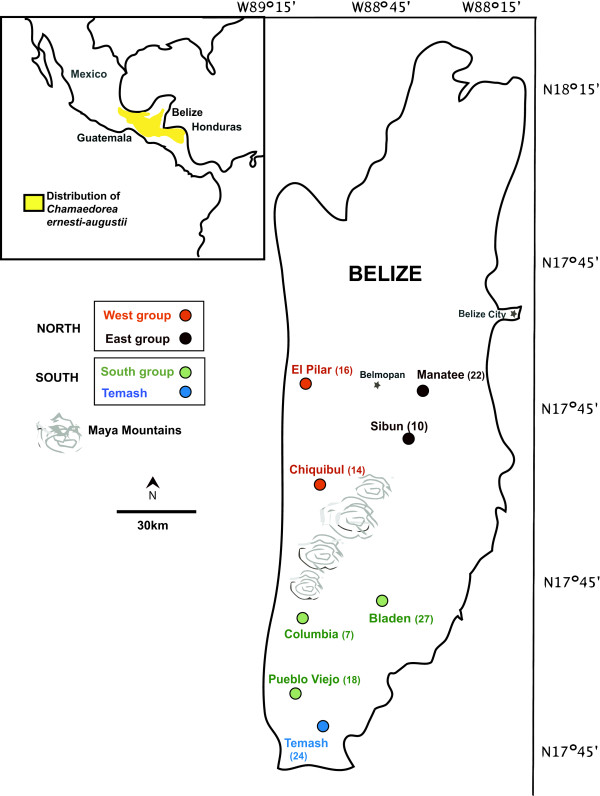
**Sampling sites in Belize**. Predicted groups based on Holocene climate change (South and South regions) and Mayan deforestation (west-east group and South group) with Temash as an outlier are show in gray.

This study focuses on eight localities of Belizean *Chamaedorea ernesti-augusti *(Figure 2). In Belize the presence of various wild populations have, until recently, remained largely undisturbed by human harvesting practices. Its primary habitat consists of wet to dry lowland tropical broadleaf forests, locally disrupted by the occurrence of drier forests in the rain-shadow of the Maya Mountains and mostly absent in the far north of Belize [40]. It is typically found on limestone-enriched soils in lowland and submontane broadleaf forests. Belizean forests provide an appropriate scenario in which to test the effects of historical and contemporary habitat changes on the distribution of genetic variation of *C. ernesti-augusti*. Early Holocene climate change impacted considerably on the ecology and species composition of American landscapes [41-44], including Belize. Pine savannahs and tropical mesic forests spread after an abrupt increase in temperature and rainfall in Belize during the early Holocene at 10,000 BP [45,46]. Belizean forests were then impacted by Mayan land use for *ca*. 1,500 years [41,47]. Areas of significant Mayan disturbance include the highly fertile Belize River Valley [48], forest-covered terraces in the Chiquibul cultivated by a population of 120,000 people [49,50], the upland areas to the north (El Pilar and southeast of El Pilar) and the south, near Pueblo Viejo [48]. Following the Mayan collapse, forests regenerated rapidly over most of the country but were affected again when extractive logging began in the 1700s [51]. Approximately 22% (7,200 ha) of Belize's forest has been lost since 1989, with deforestation rates recently escalating [52].

Our objectives are to test specific hypotheses about the evolutionary history of *C. ernesti-augusti *and to establish a genetic baseline for its management and conservation. We measure the levels of genetic variation and genetic structure of 138 individuals of *C. ernesti-augusti *throughout Belize in eight localities, using nine nuclear microsatellites. We describe genetic patterns within and among populations and test whether two major landscape events have shaped the current population structure of *C. ernesti-augusti *in Belize. These events are major climatic, vegetation and soil changes during the early Holocene (*ca*. 10,000 BP), and the impact of Mayan and recent landscape fragmentation, including aridification during Mayan decline (*ca*. 1,000 BP). We used traditional summary statistics and the theory of the coalescent as proxies for the historical effects on genetic structure of *C. ernesti-augusti *in Belize. Bayesian statistics were used to yield an *a posteriori *partitioning of the contemporary genetic variation (*i.e*. within the last two generations), and to test the assumed subdivision caused by deforestation within the northern and southern regions.

This study is one of few addressing the genetic structure of wild populations of ecologically and economically important understory palms, and the first to use highly variable molecular markers in this genus. We discuss the distribution of genetic variation within and among populations of *Chamaedorea ernesti-augusti*, their interplay with putative historical and contemporary geographic events, and their implications for conservation.

## Results

### High allelic richness and high inbreeding

**Figure 3:**
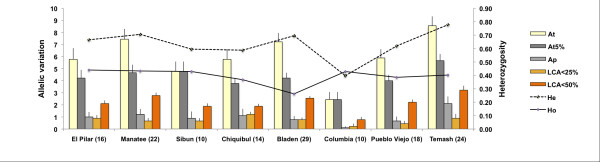
**Allele patterns for nine microsatellite loci in Belize**. *At *is the number of alleles, *At5*% is the number of alleles with frequencies above 5%, LCA are the least common alleles (less than 25%), *Ap *is the number of private alleles. Observed and unbiased expected heterozygosity *Ho *and *He *respectively [113], are shown for each locality. Sample sizes are given in parentheses.

We sampled eight *C. ernesti-augusti *sites throughout Belize with an average of 17 individuals per locality (Figure 2). For the most part our sampling reflects the natural disjointed distribution of *C. ernesti-augusti *in Belize, and its low population densities compared to other sympatric congeners [53]. Linkage disequilibrium was observed in some localities, the most significant being Pilar and Temash, in loci 16A2-AD1 and 15C2-22D8 loci, respectively. Significant deviations from HWE (*p *< 0.01) were observed in 21% of loci, but there was no apparent pattern by locus or site. The mean number of alleles across all populations was *t *= 6.9 (CI_95% _± 1.87). Figure 2 shows the allelic patterns across populations and levels of heterozygosity. The mean total number of alleles per population was highest in Temash, *At *= 9.00 (SD = 2.46) and lowest in Columbia, *At *= 2.86 (SD = 1.75; Figure 3). The limited allelic diversity found in Columbia could be due to the fact that this is our locality with the smallest sample size (N = 7). However, this region also has the least abundant populations of wild *C. ernesti-augustii *compared to densities from the Chiquibul Forest Reserve or other areas in Belize (Table 1) [41]. Columbia has been greatly fragmented despite its protected status, and has been affected by both fires and hurricanes [37,54]. It is also one of the main sites with recent harvesting [37] and should be followed-up in future studies. Across populations, the average heterozygosity was *He *= 0.67 (CI_95% _± 0.06) and *Ho *= 0.39 (CI_95% _± 0.049). Bladen and Manatee have the lowest and the highest observed homozygosity, respectively (Figure 3). Temash had the highest number of private alleles (*A*_*P *_= 1.75).

**Table 1:** Sampling sites

**Site**	**Site Area (Ha)/conservation status^1^/connectivity^2^/harvested^3^**	**Alt^4^(m)**	**N/ha^5^**
**El Pilar (16)**	50/core/isolated/yes	0-300	650
**Manatee (22)**	103,907/core/restricted to soil type + narrow valleys/no	0-200	650
**Sibun (10)**	19,000/core/restricted to one small site/no	0-200	--
**Chiquibul (14)**	59,822/core/continuous/yes	400-700	200
**Bladen (27)**	40,485/core/continuous/yes	0-200	400
**Columbia (7)**	60,000/protected/fragmented/yes	0-300	47
**Pueblo Viejo (18)**	15,000/unprotected/isolated/no	0-300	225
**Temash (24)**	16,945/buffer/isolated/yes	0-200	200

**1-**Conservation status is based on the current classification of the area within Belize's Natural Areas system. Core corresponds to the protected zone of the reserve. Buffer areas are outside the main core or are unprotected at the federal level **2- **Connectivity refers explicitly to the continuity of area where *C. ernesti-augusti *is found. **3- **Harvest is based on observation and personal communication with the owners of the land. **4**- Altitude; **5- N/HA **is number of individuals per hectare. Samples sizes per locality are in parenthesis.

### Dating and quantifying changes in effective population size

**Figure 4:**
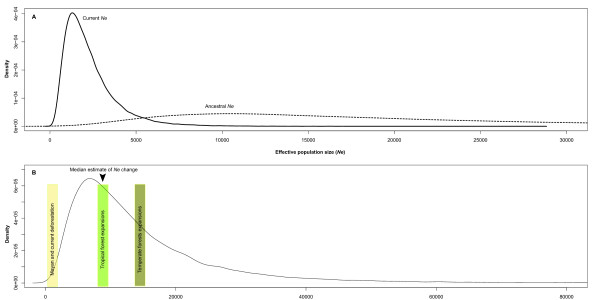
**MSVAR estimates of changes in effective population size**. Quantification and timing of a population bottleneck in *C. ernesti-augustii*. A) Estimated changes in effective population size, with the current *Ne *(*N*_0 _= 1,862) smaller than the ancestral *Ne *(*N*_1 _= 16,000). B) The posterior distribution of the time since the population bottleneck. The median is 10,715 years ago (arrow), coinciding with a major climatic change that took place at the end of the Pleistocene (*ca*. 10,000 ya) There is no evidence of a population bottleneck closer to period of Mayan deforestation (*ca*. 1,000 ya).

High levels of inbreeding and low genetic variation are in part, a result of major historical and contemporary landscape changes. We tested the effects of climate change and deforestation on recent and historical effective population size. The distribution of allele frequencies based on the Luikart *et al *[55] graphical method did not reveal recent bottlenecks. The coalescent-based method MSVAR based on [56] provided information on the historical changes in effective population sizes (*Ne*), and their approximate timing. Independent runs with varying assumptions on microsatellite mutation rates (m) gave congruent results, the most stable estimate being m = 10^-3^. All of the five MSVAR analyses completed (m = 10^-3^; generation time = 10), consistently uncovered a population decline from an ancestral *Ne *of approximately 16,000 (HPD_95% _= 1,738-5,104; mean = 2,059 median = 1,621) to a current *Ne *of 1,862 (HPD_95% _= 301-5161; mean = 2,236; median = 1857; Figure 4a). We selected the medians and the lower and upper limits of the 95% highest posterior density (HPD_95%_) as proxies for the time in generations and the amount of change in population size, because when distributions are skewed, medians can be better descriptors than the standard arithmetic mean. The distribution of the time in generations since the population started to decrease had a median of approximately 10,715 years BP (HPD_95% _= 1,245-3,627; mean = 14,120; Figure 4b). There was no evidence of more recent change in effective population size during the Mayan deforestation.

### Genetic connectivity across Belize

Using the eight sampling sites as populations, the estimate of genetic structure *θ*_ST _indicated that a moderate 8.5% (CI_95% _0.05858-0.11663) of the variation resides among localities. According to AMOVA, most of the variation is found within individuals, and among individuals within a single population, rather than among populations within each region (see Additional file 1). Fixation indices suggest that there are high levels of inbreeding, with the variation within individuals relative to the subpopulations as *F*IS = 0.384 (*p *< 0.01) and within individuals relative to the total as *F*IT = 0.425 (*p *< 0.01).

**Figure 5:**
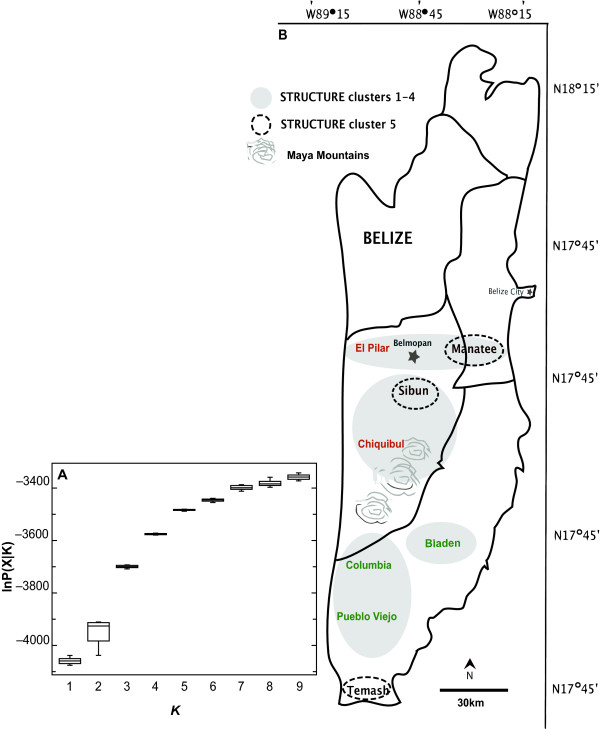
**Five genetic clusters according to STRUCTURE**. Shown are the most likely number of clusters (*K *= 5) estimated without *a priori *information on population origin. A) Box plots of the likelihood across lnP(X|*K*) across numbers of clusters. The line inside the box represents the median of the likelihood distribution, the box lower and upper edges represent the 25^th ^and 75^th ^percentiles, respectively, the bars represent the 5^th ^and 95^th ^percentiles, and open circles are far outside values. B) Map showing distribution of each genetic cluster based on admixture values and the likelihood of *K *= 5.

By employing individuals and landscapes in probabilistic and likelihood analyses, Bayesian methods have provided tools for different levels of spatial and temporal resolution to population-level studies [57]. When no *a priori *information is provided on the geographic origin of each population in STRUCTURE, the lowest marginal likelihood and variance detected *K *= 5 (Figure 5a), while the Evanno *et al *[58] Δ*K *method favored both the model of *K *= 3 and *K *= 5 (Figure 5b). When geographic information was included the optimal partitioning was *K *= 5 (Additional file 2). The two feasible estimates (*K *= 3,5) with STRUCTURE and the extent of LD support the notion of high genetic connectivity across Belize, yet with some degree of differentiation. Localities are somewhat clustered into the expected north and south division, but there are different levels of connectivity within localities and some outliers such as Sibun and Temash.

**Figure 6:**
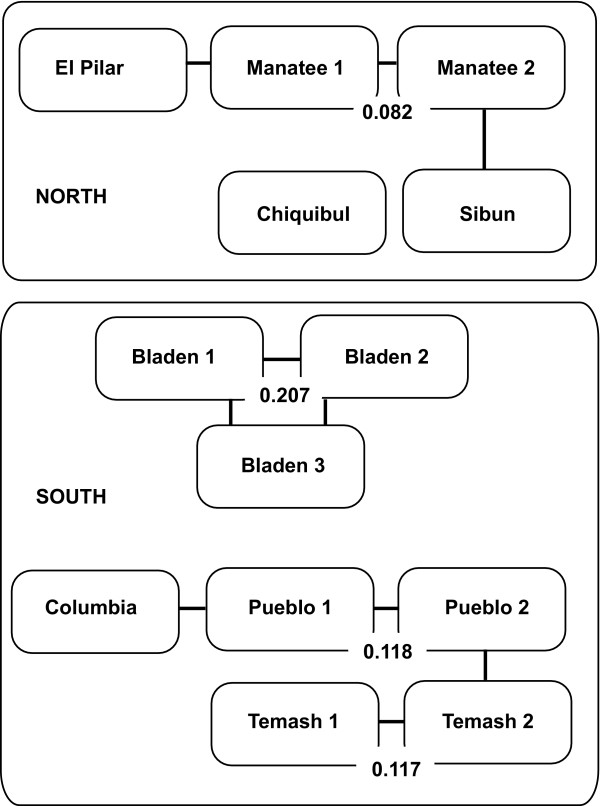
**Inferred subpopulations within localities using STRUCTURE hierarchically**. The name of the locality is shown in a box, subpopulations are indicated with numbers, and lines connect subpopulations that have shared migrants. Differentiation within localities with subpopulations detected with STRUCTURE was calculated by *θ*_ST _[115]

When the populations from the north and south were analyzed independently of one another, five and eight genetic clusters were identified, respectively (see Additional file 3). We also assigned individuals based on possible geographic barriers to migration, and identified those sampling sites that were divided into subpopulations. In both cases, El Pilar and Sibun have some degree of admixture with Manatee, but appear to be single populations. Manatee is subdivided into two genetic clusters with weak differentiation (θ_ST _= 0.082) among them; one has migrants shared with Sibun based on the inferred ancestry of individuals, and the other has mixed ancestry with El Pilar. Chiquibul has a few migrants from Bladen, but when compared with the rest of the northern sites, it segregates into a distinct population. Bladen is composed of three genetic subpopulations with high differentiation among them, and a small portion shared with Pueblo Viejo. Temash is divided into two genetic clusters, with one of the clusters sharing membership with Pueblo Viejo, and Pueblo Viejo sharing half of its individuals with Columbia. Both Pueblo Viejo and Temash have moderate differentiation among their subpopulations (Figure 6).

In summary, there are subpopulations with various degrees of differentiation within some of the localities sampled. Shared migrants among particular regions result in overall high genetic connectivity across the country.

### Pairwise immigration rates among populations

To investigate migration rates, we estimated the proportion of migrants among sampling localities using BayesAss (Table 2). To control for sampling size differences across Belize, and for populations that were potentially missed in our sampling scheme ('ghost'), we used GeneClass (Table 3). This implementation is also useful when sample sizes are relatively small, which is the case for some our localities (e.g. Sibun, Columbia). Given the high levels of gene flow, sampling localities could be used as populations (instead of inferred genetic clusters). The significant χ^2 ^values and log-likelihood ratio tests (*p *= 0.001) excluded the possibility of a lack of sufficient microsatellite polymorphism to detect migration rates between sampled localities in BayesAss. Both analyses failed to identify regional structure, but suggested higher exchange within northern and southern regions rather than between them. Most individuals were assigned to the same Bayesian clusters estimated in STRUCTURE (Additional file 1).

**Table 2:** Bayesian assessment of migration within and among sampling localities implemented in BayesAss+.

	**Sampling Locality**							
	
	**North**				**South**			
	**PIL**	**MAN**	**SIB**	**CHI**	**COL**	**BLA**	**PVI**	**TEM**
**PIL**	**0.980**	0.255	0.142	0.154	0.130			0.079
	(0.019)	(0.035)	(0.057)	0.077	(0.076)			(0.046)
**MAN**		**0.684**						
		(0.017)						
**SIB**			**0.699**					
**CHI**				**0.688**				
				(0.020)				
**COL**					**0.713**			
					(0.041)			
**BLA**						**0.678**		
						(0.011)		
**PVI**				0.110	0.052	0.294	**0.977**	
				(0.075)	(0.062)	(0.022)	(0.020)	
**TEM**			0.072					**0.867**
			(0.049)					(0.052)

For each sampling locality, numbers are the mean proportion of individuals from each source locality. Bold face terms along the diagonal are proportion of non-migrants (self-recruitment). Standard deviation is given in parentheses. Empty cells represent mean proportions of lower than 0.050. PIL-El Pilar, MAN-Manatee, SIB-Sibun, CHI-Chiquibul, BLA-Bladen, PVI-Pueblo Viejo, TEM-Temash. Inner squares represent the North and South division in Belize.

**Table 3:** Assignments for each individual across populations according to GeneClass.

	**North**				**South**			
**Assigned to**	**PIL**	**MAN**	**SIB**	**CHI**	**COL**	**PVI**	**BLA**	**TEM**
**PIL**	**0.427**							
**MAN**		**0.416**		0.068		0.137		
**SIB**		0.127	**0.434**			0.077		
**CHI**	0.087	0.192		**0.491**	0.512	0.088	0.064	0.085
**COL**		0.240		0.115	**0.638**	0.125		0.094
**PVI**						**0.268**		
**BLA**							**0.304**	0.083
**TEM**		0.141	0.074	0.136		0.166	0.153	**0.404**

**Assignment proportions**	**PIL**	**MAN**	**SIB**	**CHI**	**COL**	**PVI**	**BLA**	**TEM**

**Assigned** **(sum of probabilities)**	0.51	1.12	0.51	0.81	1.15	0.86	0.52	0.67
**Unassigned** **(1-assigned)**	0.49	-0.12	0.49	0.19	-0.15	0.14	0.48	0.33

This algorithm estimates the probability that each of the plants is assigned to a particular population, depending on where its genotype is more likely to occur. Each cell represents the average probability of assignments in each locality. In bold along the diagonal are the number of residents within a population (self-recruitment). Northern and Southern clusters are highlighted. Empty cells represent mean proportions of less than 0.050 and were discounted in the assignment proportions. Proportions of assigned individuals are summarized, not all individuals are assigned to known sites (columns add to <1.0), and one individual can be assigned to more than one site (in which case columns add to >1.0). PIL-El Pilar, MAN-Manatee, SIB-Sibun, CHI-Chiquibul, BLA-Bladen, PVI-Pueblo Viejo, TEM-Temash.

Overall, BayesAss estimated about 30% of the overall individuals being exchanged with other sites (Table 2). GeneClass showed a similar pattern, with individuals successfully assigned to a known population only 20% to 40% of the time, with a slightly higher assignment rate in Sibun and Temash (54% and 60%, respectively) (Table 3). Pairwise comparisons across localities with BayesAss showed that El Pilar in the north and Pueblo Viejo in the south are mostly self-seeding, with 98% and 97% respectively of the individuals originating from within the same site. Both also contribute migrants to some of the neighbouring populations.

These analyses suggest that some areas had individuals originating from both the north and south. For example, Chiquibul has received migrants from both El Pilar and Pueblo Viejo. GeneClass showed that the Columbia population had the highest amount of migrants that originated within its boundaries, but also a high frequency of exchange with Chiquibul. The latter contrasts with the BayesAss estimates that link this population to El Pilar and Pueblo Viejo. This is probably due to the small size of the Columbia population, which can lead to biases in migration estimates calculated in BayesAss. An accurate estimate of number of individual migrants will likely arise when all potential 'ghost' populations are sampled, but both GeneClass and BayesAss provide a finer picture of immigration rates.

### Isolation by distance

Significant isolation-by-distance was only detected within a subset of populations in the northern region from El Pilar, Manatee and Sibun (r_*xy *_= 0.168, *p *< 0.05), a region with directional migration as detected by BayesAss. Comparisons across all sampling localities and the subregions identified with STRUCTURE or directional migration patterns between Pueblo Viejo and other sites had no meaningful patterns of isolation by distance.

## Discussion

### Within-population patterns: allele rich but highly inbred species

*Chamaedorea ernesti-augusti *has a mixed pattern of moderate to high allelic diversity and high inbreeding. The number of alleles per locus across sites is congruent with expectations for perennial, outcrossing species [14], and slightly lower than other palm species assessed with microsatellites, such as *Euterpe edulis *(*t *= 10) [59] and *Phoenix atlantica *(*t *= 8), which are both canopy palms [60]. Deviation from Hardy-Weinberg equilibrium in *C. ernesti-augusti *is caused by an excess of homozygotes from increased inbreeding, rather than null alleles. Inbreeding estimates in *C. ernesti-augusti *with respect to subpopulations (*F*IS), and relative to the total (*F*IT), are higher than values reported for other palms with microsatellites [10,59,61], which have levels similar to those of other rainforest species [1,62].

In most plants, the combination of low heterozygosity and high inbreeding is a result of mating and demographic factors that increase selfing and nonrandom mating between closely related individuals [e.g. [7,63]]. In *C. ernesti-augusti *these patterns are most likely a result of pollination by *Brooksithrips chamaedoreae *thrips and demographic dynamics within populations. Despite a generalized notion in which thrips are not perceived as effective *Chamaedorea *pollinators [64-66] but see [67], thrips are the main pollinators of *C. ernesti-augusti *and three other sympatric congeners in Belize [30]. Thrips have a limited dispersal range of only a few meters, therefore increasing the chances of inbreeding by landing among plants that are in close proximity to one another. Although they are limited travellers, thrips are very efficient pollinators in other plant species [68-73] and can direct their flight towards certain inflorescences [74,75]. In Belize, clouds of *B. chamaedoreae *pollinate *C. ernesti-augusti *in a dependent mutualism in which female and male thrips receive a diverse array of rewards in both staminate and pistillate flowers [30]. The large number of thrips moving among plants of *C. ernesti-augusti *transport pollen amounts comparable to other known pollinators, sufficient enough to enact pollination and subsequent fruit set [30]. While restricted movement from thrips could result in inbreeding, its efficiency in pollination could simultaneously maintain allelic richness. Even more so if different pollen donors contribute in different years, or if thrips move among different groups of inflorescences [30]. Specific studies on pollen versus seed-mediated gene flow will help test these hypotheses in more detail.

Complex demographic patterns within populations, such as sex-biased distributions and differences in flowering times within populations, can also result in high levels of inbreeding [7,76]. *Chamaedorea *and other palm genera have local aggregations of specific cohorts or sexes [33,77-80]. Although the distribution of nearest-neighbours of staminate and pistillate flowers in *C. ernesti-augusti *species is random with respect to one another, staminate and pistillate individuals are significantly aggregated at the community level [30]. If there is mating-pair heterogeneity, or if floral resources from either females or males are not available in given years, the number of genetic donors per generation could be decreased. Local spatial distribution of sexes can lower reproductive success, decrease observed heterozygosity, and in some cases increase local differentiation [65,81,82]. The reduced rates of heterozygosity observed in *C. ernesti-augusti *may also be a consequence of reduced fruit set observed in some localities within Belize or the result of female plants having restricted fertility patterns [30]. Two other *Chamaedorea *species, with a similar genetic pattern to *C. ernesti-augusti*, have females that only reproduce for a few years within their life cycle (*C. elatior*) or have populations with a subset of females dominating successful reproductive events (*C. tepejilote*) [7].

Demographic bottlenecks decrease local survival in other *Chamaedorea *palms (e.g. *C. tuerkheimii *and *C. elegans*; [83]) and could further influence patterns within populations in *C. ernesti-augusti*. Tree falls, common predation by ground moles, and increasing harvesting by *xateros *increase adult mortality in this species, reducing the number of reproductive individuals and decreasing overall survival at the community level [30]. This species generally occurs at densities of less than 100 individuals per hectare in some areas (e.g. the Greater Maya Mountains), and at the scale of a few hundred individuals per hectare for most of its distribution [53]. Most interestingly, its distribution across Belize is not homogenous, its density and local aggregation varying considerably [37,84]. Low density of *C. ernesti-augusti *within subpopulations could further limit pollen movement by reducing the number of donors, and increasing susceptibility to genetic drift by reducing local effective population sizes. The highest number of private alleles and overall allele diversity is indeed found in localities with large contiguous populations (e.g. Chiquibul and Temash).

### Genetic signature of a population decline: changes in effective population size driven by Holocene forest expansions

If recurrent landscape changes were indeed a predominant barrier to gene flow of *C. ernesti-augusti *populations, genetic structure through most of the Holocene would have created a southern and a southern division, with the far southeast, which has a distinct geological and geographical history, forming either an outlier or part of the southern region. Climatic change during the late Pleistocene and early Holocene have influenced the population genetics of other taxa [85]. We know that changes in forests after the last glaciation in Central America were far from linear [41], but less is known about the migration and subsequent interaction of understory species. We detected a reduction in effective population size (*Ne*) that coincides with major climatic and landscape changes in Belize and neighbouring regions in the early Holocene [45,46]. Prior to this time, the climate was probably too cold and/or dry to support *Chamaedorea *palms in the region [41]. The genetic reduction of its effective population size at 10,715 BP suggests that *C. ernesti-augusti *migrated into Belize when tropical forests expanded at 10,000 BP [86]. Its current absence from suitable limestone soils in the south of Belize due to low rainfall supports the hypothesis that this species may have migrated into this area only when humidity increased, creating a founder effect around this time. Arid episodes during the Holocene in this region probably led to reduced heterozygosity and the loss of rare alleles as population numbers and sizes decreased.

Long-distance seed dispersal has likely played an important role in *Chamaedorea *species that moved into Belize. Although most of the theoretical and empirical evidence of long-distance seed dispersal events comes from trees [5,87-90], this mechanism probably occurred in other understory species during the Holocene [91]. As Cain *et al *[91] suggested, it is likely that species with the life history characteristics of *C. ernest-augusti *have a stratified dispersal model, where local movements occur by one set of mechanisms (e.g. thrip pollination and local seed dispersal by mammals) and accidental or sporadic long-distance transport (e.g. dispersal by birds) occur by another.

### No evidence of Mayan influence or recent fragmentation effects

Massive deforestation by the Ancient Maya over a period of 1,500 years in the same region probably reduced all western *C. ernesti-augusti *populations found in the understory of closed mature tropical broad-leaf forests within Belize, as well as other congeners, except *C. seifrizii*, which has a different distribution pattern. The impact of Mayan deforestation would result in a more recent bottleneck and yield a second subdivision within the southern region. Populations along the border with Guatemala have also recently decreased as a result of fragmentation and poorly regulated harvesting in recent years.

Population genetics theory in tropical species predicts that restricted pollen movement or human-induced barriers among populations can create an isolation-by-distance pattern [92,93]. It also predicts that genetic erosion driven by drift could eventually decrease the overall genetic connectivity of the species in a fragmented landscape [94]. Most forest species are indeed perceived as genetically vulnerable following human-induced fragmentation, a particularly acute problem for wild-harvested species, whose populations can suffer additional reductions as a result of over-harvesting [95]. Yet, although *C. ernesti-augusti *has low heterozygosity, and has experienced at least one major reduction in its effective population size, there was no evidence of a recent mode-shift in allele frequencies across all loci in Belize patterns expected after significant changes in *Ne *[55]. We did not detect changes in the past few thousand years with MSVAR (Figure 3). There is also high regional genetic connectivity and little evidence of isolation by distance throughout Belize.

There are several possible explanations for these patterns. Most likely, founder effects during the early Holocene may be confounding the detection of more recent demographic changes. Simulations have shown that genetic diversity can be maintained at the landscape level after a major founder effect [96], for instance if there is a combination of multiple seed-driven small founder effects that result in different maternal lineages, followed by pollen-mediated gene flow [i.e. [97]]. Diverse land use patterns during Mayan deforestation may have helped mitigate the impact of habitat degradation and forest fragmentation [41], allowing populations of *C. ernesti-augusti *to survive and re-expand locally. In combination with occasional long-distance seed dispersal, overlapping generations in *C. ernesti-augusti *could also provide sufficient time and opportunity to buffer more recent biases in allele distribution at the regional level (e.g. [98,99]).

There is in fact, a relatively limited amount of empirical evidence of the effects of recent fragmentation on plant species [15]. Some Neotropical and temperate plants have shown unexpectedly high levels of gene flow, in a few cases as a result of increased propagule movement after habitat deforestation [e.g. [98,100,101]]. Although there is no evidence of increased gene flow after fragmentation, the extent of the genetic neighbourhood and the distribution of allele frequencies within subpopulations of other *Chamaedorea *species (e.g. *C. elatior *and *C. tepejilote *in Mexico [7]) also supports the hypothesis that dispersal in *C. ernesti-augusti *and congeners is probably broader than previously assumed. Given that in most plants seed-mediated gene flow appears to contribute more to genetic exchange between populations than does pollen-mediated gene flow [102], this may be a critical factor for mitigating genetic bottlenecks or founder events. Additional studies in *C. ernesti-augusti *and other understory palms will help determine the exact evolutionary mechanisms behind these patterns, if long-distance seed dispersal indeed plays a major role, and if understory species are in effect as resilient to forest fragmentation as it has been suggested for canopy species [14].

### Ongoing genetic differentiation: five highly connected genetic clusters across a north-south gradient

Despite overall gene flow across Belize, within-population dynamics increase inbreeding and create subdivision at a finer spatial scale, generating recent, ongoing differentiation within localities. Our results suggest that populations of *C. ernesti-augusti *can be roughly divided into five genetic clusters with a north to south gradient. Subdivisions largely agree with expectations of landscape changes during the Holocene (*i.e*. no subsequent division of El Pilar-Chiquibul and Sibun-Manatee after Mayan deforestation). The hierarchical analysis in STRUCTURE (Additional file 1) and the immigration matrices with BayesAss and GeneClass (Table 2) revealed varying degrees of gene flow within Belize. It is worth mentioning that our estimates of differentiation based on *θ*_ST _simply provide a reference to the degree of gene flow within those subpopulations. They show that there are various degrees of differentiation throughout Belize, but given the small size of each subpopulation, do not intend to provide a genetic neighbourhood or an average estimate of gene flow for the species. The different assumptions in BayesAss and GeneClass highlight the importance of taking into account sample size and 'ghost' populations. In the case of *C. ernesti-augusti*, a species with high gene flow and relatively small sample populations, GeneClass is probably more representative of actual immigration rates.

Subpopulations with varying degrees of differentiation reflect the distribution and density of this species, which varies throughout Belize [37], and for the most part correspond to contemporary geographic and ecological barriers. In the southern region, El Pilar was previously connected to Chiquibul through extensive forests on limestone soils, although contemporary forest corridors into the Vaca Forest Reserve may maintain some gene flow across the region. The high self-recruitment values within El Pilar are possibly due to recent human settlements isolating the reserve and a west to east decrease in annual rainfall that could restrict plant survival. The acidic soils of the large expanse of the Mountain Pine Ridge and the Maya Mountains may prevent direct connections between populations in the Chiquibul versus Manatee and Sibun to the southeast, and the Bladen and Columbia Reserves to the southeast. Given the association of *Chamaedorea *to specific local environmental conditions, variations in soil composition within each site could effectively function as fine-scale geographic barriers as they do for other plants studied [103].

In the south, *C. ernesti-augusti *occurs in small and fragmented limestone outcrops in Bladen, the locality in Belize with the highest local differentiation (*θ*_ST_). Columbia is connected to southern areas in the Bladen Reserve, following a limestone continuum that is disrupted towards the rest of the southern populations, but there is no evidence from either BayesAss or GeneClass of recent migration between these populations. As with Chiquibul, Pueblo Viejo and its forest extension into Guatemala have been affected by slash-and-burn agriculture during the past 30 years. Currently only pockets of forest remain, perhaps pressuring dispersers to migrate towards larger forest remnants. Interestingly, Temash has the highest numbers of alleles, a relatively high number of private alleles, and a high self-recruitment rate. It is one of the most isolated localities geographically, and also the wettest part of Belize, but acidic soils form a continuum on the east coast. The genetic connections between Temash southern sites are not yet clear, but it is possible that Mayan populations left Temash undisturbed [41,47], thus allowing allelic richness to increase over time. It is also possible that these patterns are a result of the different geological and climatic history in Temash, which was relatively more humid than the rest of Belize during the early Holocene [41]. A genetic assessment of Guatemalan populations could help elucidate the role of Temash (e.g. possible source of migrants during arid periods) in *C. ernesti-augusti *history within Belize.

### Conservation implications

Given the genetic pattern of high local inbreeding, and the potential importance of connectivity and migration through long distance dispersal in *C. ernesti-augusti*, it is most critical to maintain population sizes and allelic diversity within populations. One important step toward the conservation of *C. ernesti-augusti *is the enforcement of the existing harvesting rate regulations from the Belize Forest Department, which specify one leaf cut per individual per year [104]. Sustained higher rates may increase mortality and decrease reproduction [104]. Decreasing the number and sources of genetic donors within populations could result in inbreeding depression or other negative short-term consequences. If seed dispersal is indeed as important as we have hypothesized for the long-term survival of this species, excessive seed harvesting could significantly decrease gene flow among localities and could erode the existing genetic variation of this species. Aggressive and widespread seed collection to replenish emerging nurseries could exacerbate the situation, especially if seed continues to be massively collected from the same populations year after year. As *Chamaedorea *species become locally extinct due to overharvesting [105,106], which has adversely affected *C. ernesti-augusti *in some parts of Guatemala [107], documenting genetic variation becomes increasingly important. In particular, understanding fine-scale genetic patterns could help tailor the management and long-term maintenance of nurseries and living collections of this important group.

## Conclusion

*Chamaedorea ernesti-augusti *is characterized by a combination of high levels of regional gene flow, moderate to high allelic richness, ongoing local genetic differentiation, and low levels of heterozygosity. These patterns are in part explained by the tenfold reduction in its effective population size during climate change in Belize at *ca*. 10,000 BP, and by reproductive and demographic patterns within populations. In *C. ernesti-augusti*, high gene flow patterns with ongoing differentiation and historical changes in effective population size probably reflect the complex composition of old and new forests in Belize. We suggest that species like *C. ernesti-augusti *have probably survived recurrent habitat changes by a combination of long-distance dispersal that maintains genetic connectivity, and reproductive and demographic patterns that maintain allele richness at a local spatial scale. We hypothesize that thrips may actually be a critical driver of genetic patterns observed within populations in this genus, while seed dispersal by birds or mammals probably plays an important role across regions.

Although *C. ernesti-augusti *is inbred, inbreeding depression is probably offset over time by the overall genetic connectivity across regions, which may have allowed this species to survive deforestation patterns during the Mayan occupation of Belize. Populations in Belize can be roughly divided into five genetic groups clustered into south and south, which correspond to geographic barriers, including differences in soil composition. Although the detrimental effects of a future reduction in gene flow should not be discounted, changes in demography due to overharvesting or localized ecological degradation are more likely to be critical for preventing genetic erosion in *C. ernesti-augusti*. Characterization of the direct relationship between demographic and genetic patterns, estimates of seed and pollen-mediated gene flow, and the population genetics of Central American and Mexican populations, will offer additional insights into the effects of regional and localized environmental changes through time, and shed light into specific mechanisms, such as long-distance seed dispersal. We are currently investigating genetic patterns in congeners in Belize and *C. ernesti-augusti *in Mexico, to determine whether the patterns observed in the present study are consistent across the species and in other species of *Chamaedorea*, or are unique to Belizean populations of *C. ernesti-augusti*.

## Methods

### Sampling scheme

Eight sites representing the broad distribution of *C. ernesti-augusti *across Belize were sampled for a total of 138 adults (Figure. 2). Euclidean distances among locations range from 20 km to 70 km. Samples were collected pivotal to a specified central point in each locality. Within sites, samples were collected from an area within 1 km^2 ^with at least 3 m separating individuals.

### Genotyping and genetic variation

QIAGEN DNEasy Mini kits (Valencia, CA) were used to isolate DNA from leaf tissue. We characterized nine nuclear microsatellites to measure genetic variation following Cibrián-Jaramillo *et al *[108]. Dataset editing and formatting was done with the Excel Microsatellite Toolkit[109]. Linkage disequilibrium (LD) [110] and deviations from Hardy-Weinberg equilibrium (HWE) could indicate the presence of population structure or inbreeding [111], therefore the presence of LD was investigated at the 5% statistical significance level among loci per population based on sampling sites with 10,000 permutations. Departure from HWE expectations was tested for each locus with default parameters; both analyses were carried out in Arlequin[112]. Heterozygosity was measured as the unbiased expected heterozygosity (*He*), and observed heterozygosity (*Ho*) corrected for sample size [113]. All genetic variation estimates were obtained in Genetix v4.05 [114]. The presence of null alleles and allelic dropout was tested using Micro-checker v2.2.1 [115], which calculates the probabilities for the observed number of homozygotes within homozygote classes using a cumulative binomial distribution [110].

### Genetic signature of a population decline

If the effective population size of *C. ernesti-augusti *suffered a bottleneck or a founder effect after climate change in the Holocene and/or after Mayan deforestation, populations should show a bias in the distribution of allele frequencies. Alleles at low frequency (<0.1) would be less abundant and rare alleles would be lost, often resulting in an excess of heterozygotes [55,116]. Allelic richness was measured based on the number of alleles per population (*A*_*T*_), the number of alleles with a frequency greater than 5% (*A*_*T*5%_), less than 50% (*A*_*T*<50%_), and the number of private alleles (*A*_*P*_) [117]. The distribution of allele frequencies was estimated following the graphical method of Luikart *et al *[55].

We also examined traces of a major population change and, most importantly, the time in generations since the population size changed, using the coalescent-based Markov chain Monte Carlo method [118] implemented in MSVAR v1.3 [56]. This method assumes a single population (all eight localities of *C. ernesti-augusti *in Belize together) of size *N*_1 _that changed in size exponentially or linearly in the past (*t*_*a *_generations ago) up to the current population of size *N*_0_. The posterior distribution of the demographic and genealogical history is given by a prior distribution of demographic and mutational parameters. These are *tf *= *t*_*a*_/*N*_0_, *r *= *N*_0_/*N*_1 _and q = 2*N*_0_m, where m is the locus mutation rate, *tf *is the time at which the population started changing in size, scaled by *N*_0_, the population size at present (time of sampling), and *N*_1 _is the population size in the past. Given the lack of information on the evolution of microsatellites in palms, we varied the microsatellite mutation rate (10^-3^, 10^-4^, 10^-5^) in order to examine the congruence of posterior estimates of *N*_1 _to *N*_0_, and *t*. We also varied the generation time with five and 10 years using a mutation rate of 10^-3^. We used the exponential change model with the remaining parameters as defaults for all runs. An exponential model is more suitable for modelling population changes over a shorter time scale (e.g. as a result of a sudden expansion). Each run was conducted for 200 million generations with a 10^4^-step thinning interval.

We used a 50% burn-in before combining the posterior parameter estimates. Priors were the same across all runs except for the hyperprior means and variances for log *N*_1_. Those were established as 4.0 3 0 0.5; 3.5 3 0 0.5; 3.0 3 0 0.5; 3.0 3 0 0.5; and 3.0 3 0 0.5, for α_N1_, σ_N1_, β_N1_, and τ_N1_, respectively. In order to examine the congruence among independent runs with the same initial assumptions, we chose the scenario with m = 10^-3 ^and a generation time of 10 years, and conducted five independent runs with the remaining parameters as described. We chose 10 years to take into account the fact that not all individuals reproduce yearly, not all seeds survive and seeds are sourced from a few plants [30]. Posterior densities were plotted in R v2.6.1 .

### Regional differentiation

We estimated an overall measure of differentiation across all localities using θST[119]. To test the hypothesis that gene flow is reduced among regions that could have been historically subdivided, we measured AMOVA *f*-statistics [120] with Pilar, Chiquibul, Sibun, and Manatee grouped into the southern region, and Columbia, Pueblo, Bladen, and Temash into the southern region. These calculations were carried out in Arlequin.

STRUCTURE [121] was used to estimate the number of genetic clusters (*K*) given the sampled genotypes. All individuals were clustered first without providing information on their geographic origin. Ten independent runs were performed, with values of *K *ranging from 1 to 10, a burn-in of 200,000 generations and a subsequent 300,000 Markov chain Monte Carlo (MCMC) steps. Allele frequencies were assumed to correlate (prior mean = 0.01, prior SD = 0.05, λ = 1.0). Because we assumed that each individual draws a fraction of its genome from each of the *K *clusters, we set a uniform prior on admixture and α (initial value = 1.0, max = 10.0, SD = 0.025) as recommended by [119]. Admixture and allele frequency correlation are viewed as valid assumptions in the case of subtle population structure (e.g. *F*ST <0.05) [122]. A second run with the same parameters was performed providing *a priori *information on geographic origin, and a third hierarchical analysis run was used to test the most likely number of clusters within each expected north and south region, until a single cluster was found (*K *= 1). *θ*_ST _was calculated within localities that had subpopulations. In all sets of runs, the most probable number of genetic clusters was determined from the approximation of the posterior probability for different values of *K *from independent runs for each *K*. The lowest log probability of the data Pr (X|*K*), in combination with the smallest variance for each value of *K*, was used as a measure of the most likely number of clusters, as recommended by [119]. The second-order rate of change of the log probability of the data with respect to the number of clusters (Δ*K*) was also used as an estimator of the most likely number of clusters [58].

### Recent immigration among sites

We tested for recent subdivision using BayesAss+ v1.3 [123]. This method estimates a matrix of pairwise recent immigration rates among populations, *m*, using a coalescent approach. This approach assumes that the estimated clusters are a result of gene flow in very recent generations [56]. Default settings for burn-in and number of MCMC iterations were enough to reach convergence based on visual inspection of likelihood scores. Five independent runs were performed to test for congruence, and a likelihood ratio test was then employed to determine whether the prior and posterior probabilities of migration rates are significantly different from each other [123]. The presence of linkage among some loci and the overlapping generations of *C. ernesti-augusti *could decrease the accuracy of each estimate, but not necessarily change the relative proportions of *m *among localities.

Small and contrasting sample sizes may confound estimates in this method, so we compared BayesAss+ with individual assignments using the frequency criterion of Paetkau *et al *[124], implemented in GeneClass v2.0 [125]. The frequency method approximates the distribution of genotype likelihoods of the individual that will be assigned to the simulated distribution of a reference population. It takes into account type I error and reduces the amount of resident individuals being excluded, as well as incorporating the sampling variance that results from different population sizes [125].

### Isolation by distance

Limited dispersal can result in a pattern of genetic differentiation that increases with geographic distance. Isolation by distance (IBD) was tested with a Mantel test for matrix correspondence [84,126]. Genalex v.6 was used to produce a geographic and genetic distance matrix using pairwise individual comparisons, following [54] and [127]. A Mantel test with populations using [128] linearized distance Fst/(1-Fst) was also calculated on the IBD webserver available at [129].

## Authors' contributions

ACJ participated in the design of the project, performed the microsatellite genotyping, analyzed the data, and wrote most of the manuscript. CDB participated in the project design, aided in laboratory work, and contributed to the writing and revision of the manuscript. NCG, SB, and RB initiated the study and participated in its design, the collection of samples, the interpretation of the results, and the revision of the manuscript. SR and MMT participated in the collection of samples, DNA extractions, and preliminary experimental protocols. DB guided and developed preliminary laboratory protocols and contributed to the writing and revision of the manuscript. WJH and RD provided financial support, laboratory facilities, and assisted with interpretation of the results.

## Supplementary Material

Additional file 1 **AMOVA in south and south regions**. AMOVA [118] among regions. Pilar, Chiquibul, Sibun, and Manatee were grouped into the Northern region, and Columbia, Pueblo, Bladen, and Temash into the Southern region. Click here for file

Additional file 2 **Graphical output of Structure for *K *= 5**. The most likely number of clusters estimated without a priori information on population origin. Results for five genetic clusters (*K *= 5) from a run of *K *= 1 to *K *= 10. Bars represent the proportion of assignment of each individual to a genetic cluster (membership coefficient Q). Click here for file

Additional file 3 **Genetic clusters estimated within south and south regions**. Box plots of the likelihood lnP(X|*K*) across numbers of clusters without *a priori *information on population origin for A) North and B) South of Belize. The line inside the box represents the median of the likelihood distribution, the box lower and upper edges represent the 25^th ^and 75^th ^percentiles, respectively, the bars represent the 5^th ^and 95^th ^percentiles, and open circles are far outside values. Click here for file
