# Supplementary material for: Population genetics of the understory fishtail palm Chamaedorea ernesti-augusti in Belize: high genetic connectivity with local differentiation
Source: BMC Genet. 2009 Oct 9;10:65. doi: 10.1186/1471-2156-10-65 (PMC2770526; doi:10.1186/1471-2156-10-65)
Supplement: Additional file 1 — AMOVA in south and south regions. AMOVA [118] among regions. Pilar, Chiquibul, Sibun, and Manatee were grouped into the Northern region, and Columbia, Pueblo, Bladen, and Temash into the Southern region. [file 1471-2156-10-65-S1.pdf]

Additional Figure 1: AMOVA in north and south regions

Source of variation	d.f.	Sum of squares	Variance components	Percentage of variation	Fixation indices
Among regions	1	7.157	-0.02160 (Va)	-0.91	-0.00912§
Among populations within regions	6	53.543	0.17936 (Vb)	7.57	0.07500*
Among individuals within populations	130	398.020	0.84969 (Vc)	35.86	0.38413*
Within individuals	138	188.00	1.36232 (Vd)	57.49	0.42512*
Total	275	646.721	2.36		

*Significant *P*-value = 0.000 with 1023 permutations; § non-significant *P*-value = 0.75

AMOVA [118] among regions. Pilar, Chiquibul, Sibun, and Manatee were grouped into the Northern region, and Columbia, Pueblo, Bladen, and Temash into the Southern region.
