# Supplementary figures and images for: Population genetics of the understory fishtail palm Chamaedorea ernesti-augusti in Belize: high genetic connectivity with local differentiation
Source: BMC Genet. 2009 Oct 9;10:65. doi: 10.1186/1471-2156-10-65 (PMC2770526; doi:10.1186/1471-2156-10-65)

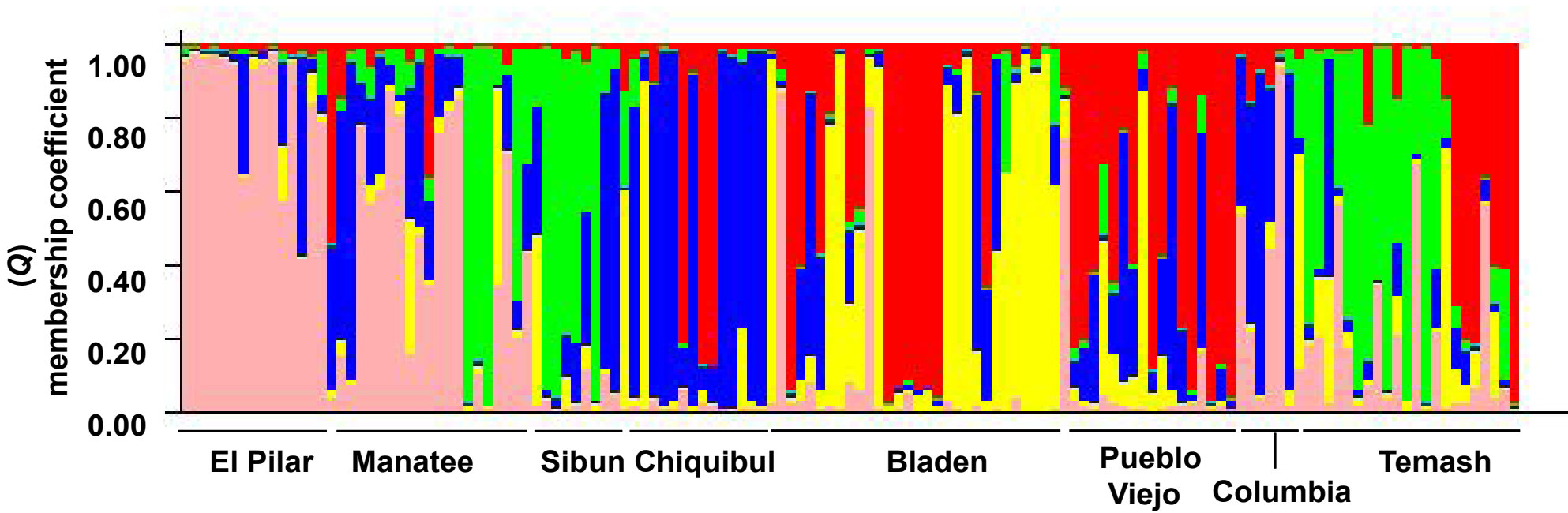

Supplement: Additional file 2 — Graphical output of Structure for K = 5. The most likely number of clusters estimated without a priori information on population origin. Results for five genetic clusters (K = 5) from a run of K = 1 to K = 10. Bars represent the proportion of assignment of each individual to a genetic cluster (membership coefficient Q). [file 1471-2156-10-65-S2.pdf]

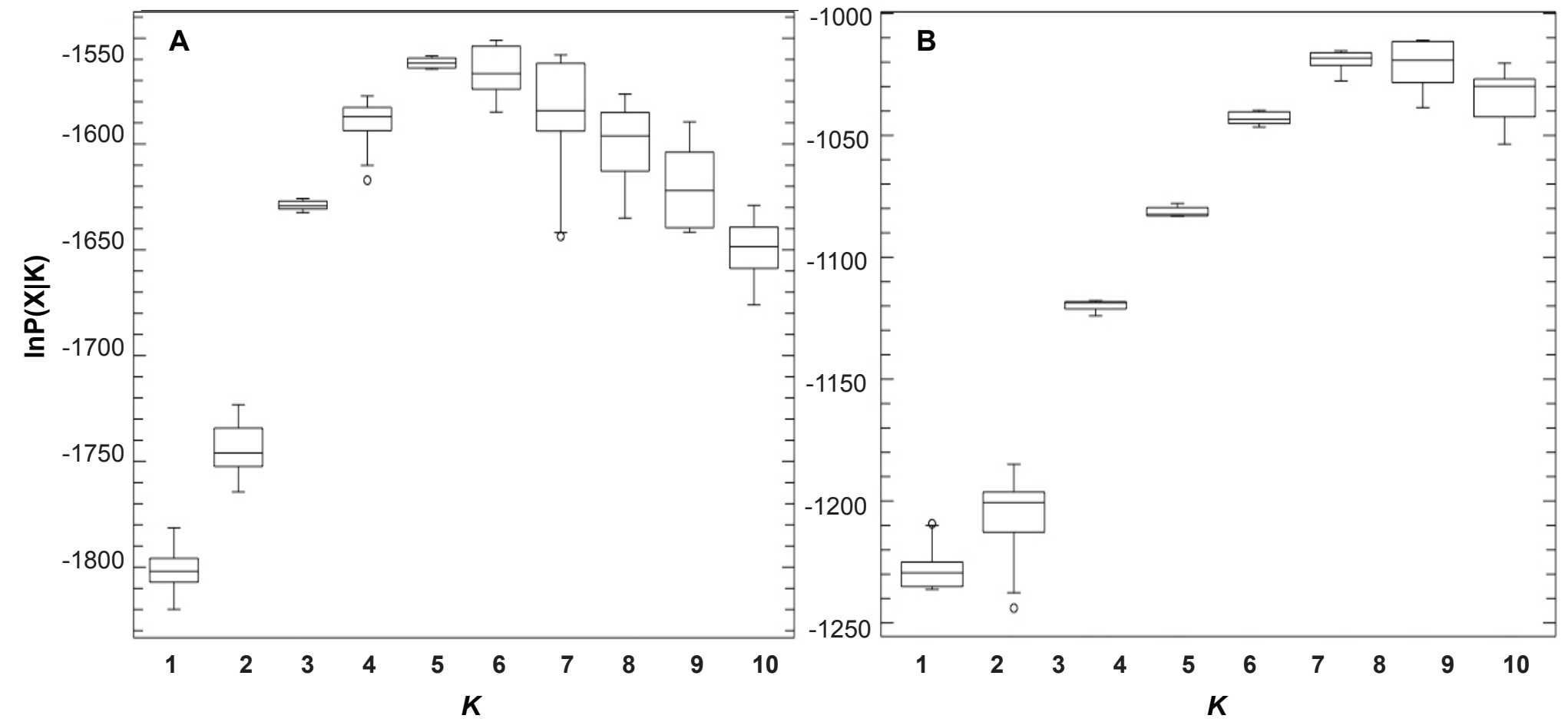

Supplement: Additional file 3 — Genetic clusters estimated within south and south regions. Box plots of the likelihood lnP(X|K) across numbers of clusters without a priori information on population origin for A) North and B) South of Belize. The line inside the box represents the median of the likelihood distribution, the box lower and upper edges represent the 25th and 75th percentiles, respectively, the bars represent the 5th and 95th percentiles, and open circles are far outside values. [file 1471-2156-10-65-S3.pdf]
